# Unusual clinical features and histopathological findings in a case of Pseudo-endophthalmitis in neovascular glaucoma

**DOI:** 10.22336/rjo.2024.34

**Published:** 2024

**Authors:** Avadhesh Oli, Agrima Bhatia

**Affiliations:** * Dept of Ophthalmology, Command Hospital (Air Force), Bangalore, India

**Keywords:** pseudo endophthalmitis, pseudohypopyon, intravitreal injections, Triamcinolone, endophthalmitis

## Abstract

**Objective:** This case report aimed to describe the unusual clinical presentation and histopathological features of post-injection endophthalmitis.

**Methods:** A 56-year-old male phakic patient with diabetic retinopathy received an intravitreal injection (Bevacizumab as per the patient) for neovascular glaucoma elsewhere and presented to our center one day after the dose with hypopyon. The eye was relatively white without pain or lid oedema. The patient was treated as a case of post-injection endophthalmitis with two doses of intravitreal antibiotics 48 hours apart. During the follow-up, he developed a Covid infection. After one week, when the media cleared, white exudates were seen in the vitreous cavity with a relatively healthy retina. He was taken up for pars plana vitrectomy and vitreous biopsy for histopathological study.

**Results:** The microscopic examination of vitreous aspirate revealed crystalline deposits without any microorganisms. Two control slides, one with a mixture of intravitreal antibiotics, which were previously injected, and the other with fresh Triamcinolone were also examined. Although the findings of the drug mixture did not match the vitreous aspirate, they matched with triamcinolone, which established it as a case of pseudo endophthalmitis due to triamcinolone injected elsewhere.

**Discussion:** Initially, it seemed like a straightforward case of post-injection endophthalmitis, but a further examination of the vitreous aspirate showed that it was pseudoendophthalmitis due to an intravitreal triamcinolone injection. Despite the patient being phakic, neovascularization or elevated intraocular pressure may have led to the disruption of the blood-ocular barrier and the migration of Triamcinolone into the anterior chamber.

**Conclusion:** The case’s uniqueness lies in being the first reported case of pseudo endophthalmitis in a phakic patient with an intact lens iris diaphragm. The case also highlighted the judicious use of available resources and out-of-the-box thinking to reach a diagnosis that may not always be obvious.

**Abbreviations:** TA = Triamcinolone acetonide, AC = Anterior chamber, IVB = Intravitreal Bevacizumab, PL = Perception of light

## Introduction

Intravitreal injections have gained popularity in the management of various retinal diseases. One of the dreaded complications is endophthalmitis and the projected incidence of acute post-injection endophthalmitis following intravitreal bevacizumab and triamcinolone is approximately 0.066% and 0.10-0.87% respectively, despite using maximal sterile precautions [**1**,**2**]. 

The incidence of non-infectious endophthalmitis after intravitreal triamcinolone has been reported to range from 0.2% to 1.6% in various series [**2**]. Pseudo endophthalmitis indicates the dispersion of drug crystals in the anterior chamber (AC) closely mimicking infectious endophthalmitis [**3**]. The benzyl alcohol preservative in the preparation has been speculated to be a stimulus for the inflammatory reaction, however, rarely, preservative-free Triamcinolone acetonide (TA) crystals causing pseudo endophthalmitis have been reported in the literature [**4**,**5**]. 

The diagnosis of pseudo-endophthalmitis is straightforward if the offending drug has been injected intraocularly, however, in this case, the patient was treated elsewhere, having a history of intravitreal Bevacizumab (IVB) injection. As per the protocol, he was initially managed as a case of post-injection endophthalmitis, however, the microscopic examination of vitreous aspirate for drug crystals unraveled the mystery.

## Material and methods

A 54-year-old male diagnosed with a case of proliferative diabetic retinopathy with neovascular glaucoma in both eyes presented to our institute with complaints of gross painless diminution of vision in the left eye. According to his history, intravitreal Bevacizumab 0.05 ml was administered in the left eye elsewhere. On examination, the BCVA of both eyes was Perception of light (PL) +.

**Fig. 1:**
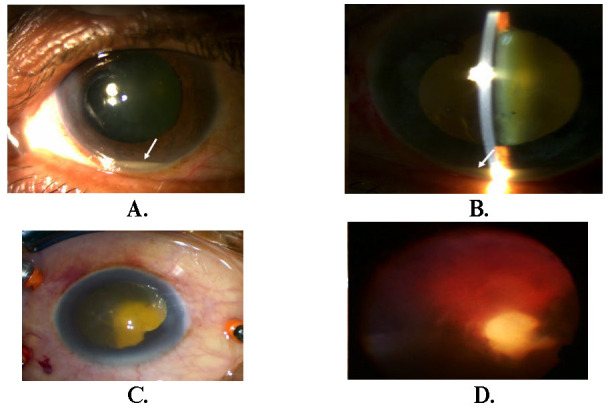
**A, B** Anterior segment findings revealing Hypopyon and 4+ cells; **C, D.** Intraoperative view, whitish exudate noted to be clumpy, intermeshed with vitreous gel, and localized without involving the rest of the retina

There was no lid edema, and the eye was white, however, anterior segment examination revealed 4+ cells and a mobile hypopyon (**Fig. 1 A, B**). The fundus glow was poor, and the fundus details were hazy. Intraocular pressure was 28 mmHg. Ocular ultrasound B Scan revealed clump-like vitreous echoes. A provisional diagnosis of acute post-injection endophthalmitis in the left eye was made. As the patient was practically one-eyed, he was planned for an anterior chamber paracentesis followed by administration of intravitreal Vancomycin (1 mg/0.1 ml) and Ceftazidime (2.5 mg/0.1 ml). The microbiological examination of the aqueous sample did not reveal any microorganisms. 

The patient did not complain of any pain on postoperative day 1 and his best corrected visual acuity (BCVA) was PL+ and there was no significant change in the anterior segment findings. To complicate the ease of workup and follow-up, the patient tested positive for COVID-19. He was continued on topical anti-glaucoma medication, steroids, and antibiotics. According to the protocol, the intraocular antibiotics were repeated after 48 hours as no response was appreciated clinically, and the B scan echoes persisted. The patient continued to be pain-free with a persistent decrease in visual acuity. He was extensively investigated for the possibility of endogenous endophthalmitis including a blood and urine culture, however, all the relevant test results were negative.

On day 7, the anterior chamber was quiet and the hypopyon had disappeared with the persistence of an abnormal white fundal glow with whitish clumped exudates in the superotemporal quadrant. Further evaluation was needed, as the patient’s visual acuity had not improved and a suspicious coagulum obscuring the posterior segment was present, in addition to his one-eyed status. There were two possibilities at that juncture, one was endogenous fungal endophthalmitis given the patient’s uncontrolled hyperglycemia or pseudo endophthalmitis or a drug coagulum based on focal, curdy white non-progressive nature. 

**Fig. 2:**
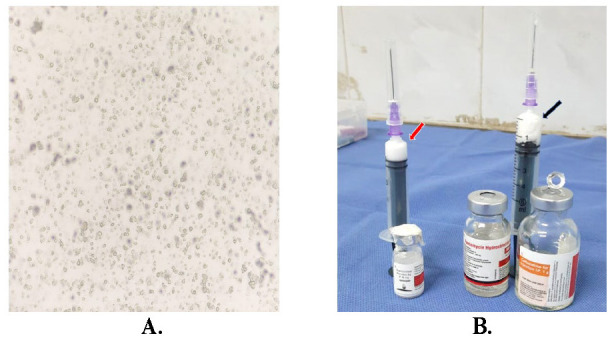
**A** Microscopy of the coagulum smear revealed well-defined, polygonal crystals; **B.** Fresh Triamcinolone (Red arrow) and another sample with a mixture of Vancomycin and Ceftazidime (Black arrow), both grossly resembling coagulum that was sent for crystal matching

To unravel the mystery, a 23 G pars plana vitrectomy with vitreous biopsy was performed to confirm the cause of the enigmatic coagulum. Intraoperatively, the exudate was noted to be clumpy, intermeshed with the vitreous gel, and localized without involving the rest of the retina (**Fig. 1 C, D**). The coagulum could be due to previous intravitreal injections, although antibiotics were given with separate syringes. The microscopy of the coagulum was done not only for microorganisms but also for drug crystals. The microbiologist was also surprised at the unusual demand. The microscopy of the smear revealed polygonal crystals (**Fig. 2 A**). 

**Fig. 3:**
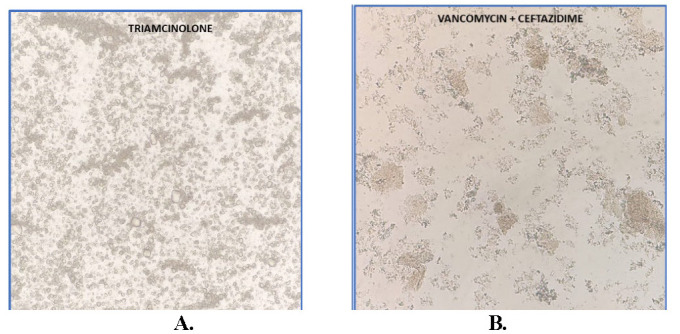
**A** The Triamcinolone sample revealed box-shaped, polygonal well-defined crystals like those noted in the coagulum; **B.** A combination of vancomycin and ceftazidime devoid of clear-cut crystals

To further characterize the crystals, triamcinolone (TA) was sent in one syringe and a mixture of 0.1 ml each of 1 mg vancomycin and 2.5 mg ceftazidime in the other for microscopic evaluation without the microbiologist knowing the contents. On Gross examination, both samples looked similar with a shiny white appearance like the coagulum (**Fig. 2 B**, red arrow TA, Black arrow antibiotic mixture). Under the microscope, the triamcinolone sample revealed box-shaped, well-defined crystals like those noted in the coagulum. The second sample of the combination of vancomycin and ceftazidime showed clusters of ill-defined structures devoid of clear-cut crystals (**Fig. 3 A, B**).

The eye remained quiet without inflammation on antiglaucoma medication. He underwent panretinal photocoagulation for proliferative diabetic retinopathy.

## Discussion

At the outset, the case appeared like a straightforward case of post-intravitreal injection endophthalmitis, however, after microscopy of vitreous revealing TA crystals, pseudo endophthalmitis attributable to intravitreal triamcinolone (IVTA) became evident. Culture-proven endophthalmitis is a potential complication of intravitreal injections. In our case, initially, there was a clear history of bevacizumab injection, and the signs justified it to be a case of post-injection endophthalmitis. The only point against it was the lack of pain, which we reconsidered and paid heed to when the microbiological investigations were negative for any growth. History of visual loss immediately or soon after injection of TA, presence of a hypopyon, anterior or vitreous inflammation, and triamcinolone crystals in the anterior or posterior chambers can aid the identification of non-infectious endophthalmitis [**2**].

We administered intravitreal antimicrobials to the patient but saw a surprising regression of hypopyon without improvement in media clarity, or BCVA. As the media cleared and we noted a persistent white coagulum, obscuring the posterior pole, we chose to do PPV with a vitreous biopsy given the patient’s gross reduction in vision. The vitreous tap was sent specially to rule out any negative fungal pathogen. We decided to explore the other end of the spectrum of pseudo-endophthalmitis. The vitreous tap revealed crystals like those of triamcinolone. The patient had also received intravitreal antibiotics, so a control slide was prepared with a mixture of vancomycin + Ceftazidime to rule out similar crystals. The histopathological study clinched the diagnosis of pseudo endophthalmitis due to TA.

Another retrospective case series including 4 patients reported an endophthalmitis-like reaction following an IVTA. There was a dense vitreous haze with a severe reduction of fundus view in all cases as was also a finding in our case [**6**,**7**]. 

Pseudo-endophthalmitis post-IVTA is a distinct clinical entity that may resolve without specific treatment [**6**]. Critical judgment by the clinician to diagnose this condition may avoid unnecessary invasive treatments. In this case, a strong clinical suspicion was the key to diagnosis despite a disconnected history. The vitrectomy and removal of TA in this case might have helped in reducing the IOP as such the patient had neovascular glaucoma.

It is well reported in the literature that anterior migration of TA can occur in eyes lacking a capsular barrier, which allows direct communication between the vitreous cavity and the anterior chamber [**8**]. Our case is unique in the sense that it is the first reported case of pseudo endophthalmitis in a phakic patient with intact lens iris diaphragm and baffled us as to how the triamcinolone injected intravitreally migrated to the anterior chamber. The possible explanation could lie in the breach of the blood-ocular barrier due to the neovascularization or raised IOP. 

## Conclusion

The case’s uniqueness lies in being the first reported case of pseudo endophthalmitis in a phakic patient with an intact lens iris diaphragm. The case also highlights the judicious use of available resources and out-of-the-box thinking to reach a diagnosis that may not always be obvious. 

**Conflict of Interest Statement**

The authors state no conflict of interest. 

**Informed Consent and Human and Animal Rights Statement**

Informed consent has been obtained from all individuals included in this study.

**Authorization for the use of human subjects**

Ethical approval: The research related to human use complies with all the relevant national regulations, and institutional policies, as per the tenets of the Helsinki Declaration, and has been approved by the review board of Command Hospital (Air Force), Bangalore, India 115/2022 dated 22 Apr 2022.

**Acknowledgments**

None. 

**Sources of Funding**

None. 

**Disclosures**

None.

## References

[1] Artunay O, Yuzbasioglu E, Rasier R, Sengül A, Bahcecioglu H (2009). Incidence, and management of acute endophthalmitis after intravitreal bevacizumab (Avastin) injection. Eye (Lond).

[2] Yoon SJ, Rhee DY, Marx JL, Blaha GR, Rogers AH, Baumal CR, Reichel E, Duker JS (2009). Anatomic and Visual Outcomes of Noninfectious Endophthalmitis after Intravitreal Triamcinolone. American Journal of Ophthalmology.

[3] Chen SD, Lochhead J, McDonald B, Patel CK (2004). Pseudo hypopyon after intravitreal triamcinolone injection for the treatment of psuedophakic cystoid macular oedema. The British Journal of Ophthalmology.

[4] Șuță MC, Karancsi OL, Mușat O, Balica N, Yasar I, Roșca C, Stanca S, Dărăbuş DM (2020). Triamcinolone acetonide induces sterile endophthalmitis in patients with intermediate uveitis: A case report series. Exp Ther Med.

[5] Mahjoub A, Ben Abdesslem N, Ben Abderrazek A, Zaafrane N, Mahjoub A, Aoun H, Jabri A, Krifa F, Ghorbel M, Mahjoub H (2022). Sterile endophthalmitis after intravitreal triamcinolone acetonide injection: A case report series. Ann Med Surg (Lond).

[6] Sutter FKP, Gillies MC (2003). Pseudo-endophthalmitis after intravitreal injection of triamcinolone. British Journal of Ophthalmology.

[7] Beer PM, Bakri SJ, Singh RJ, Liu W, Peters GB 3rd, Miller M (2003). Intraocular concentration, and pharmacokinetics of triamcinolone acetonide after a single intravitreal injection. Ophthalmology.

[8] Amato JE, Lee DH, Santos BA, Akduman L (2005). Steroid hypopyon following intravitreal triamcinolone acetonide injection in a pseudophakic patient. Ocul Immunol Inflamm.

